# Full-Duplex MAC Protocol for CSMA/CA-Based Single-Hop Wireless Networks

**DOI:** 10.3390/s19102413

**Published:** 2019-05-27

**Authors:** Yu Song, Wangdong Qi, Weiwei Zhao, Wenchi Cheng

**Affiliations:** 1 Information and Communications College, National University of Defense Technology (NUDT), Xi’an 710106, China; zhaozww@163.com; 2 National Mobile Communications Research Laboratory, Southeast University, Nanjing 210007, China; wangdongqi@gmail.com; 3 State key Laboratory Services Networks, Xidian University, Xi’an 710071, China; wccheng@xidian.edu.cn

**Keywords:** wireless networks, single-hop network model, full-duplex communication, MAC protocol, Markov chain

## Abstract

Full-duplex (FD) communication provides new opportunities for improving the throughputs of networks. However, this condition means that the number of senders increases from one to two within a certain range. We have to arrange the two nodes to send frames simultaneously in the media access control (MAC) layer. For the single-hop network model, using the FD features of the nodes and the cut-through mechanism, we propose an FD MAC protocol. The protocol improves the throughput of the network from the following two aspects. On the one hand, during the transmission of each node, based on the information of the received frame’s header, the protocol can detect collisions in the network, preventing the channel from being ineffectively occupied for a long time. On the other hand, the protocol can provide the FD with as many opportunities as possible for the nodes. According to the working process of the protocol, we modeled the states (“active” and “passive” transmission, back-off) of each node and their transitions to a Markov chain. We solved the “active” transmission probability of the node and further modeled the analytical performance of the protocol. The simulation results showed that the system throughput produced by our protocol was at least twice that of the conventional CSMA/CA protocol used in the half-duplex networks.

## 1. Introduction

Recently, with the progress in self-interference (SI) cancellation techniques, the desired reception signals of wireless nodes are no longer overwhelmed by the interference signals produced by the nodes themselves [1]. They can simultaneously transmit and receive using the same frequency band. In the physical layer, the throughput of a node working in the full-duplex (FD) mode is twice that in the half-duplex (HD) mode. It is clear that the FD mode provides new opportunities for improving the throughputs.

It has been noted that the nodes generally share the communication medium and work in a distributed manner. The traditional carrier sensing multiple access/collision avoidance (CSMA/CA) protocol has proven to be an effective media access control (MAC) protocol for the nodes to contend for the channel. Intuitively, for backward compatibility, we should apply it to the FD networks. However, FD communication means that the number of senders increases from one to two within a certain range. Originally, we only need to determine the transmission timing of one node, but now, we have to arrange two nodes to send frames simultaneously. Before sending a frame, the protocol requires that each node should sense the channel first. If it is busy, then the node cannot send the frame. In other words, when a node transmits the signal, it does not allow its neighbors to access the channel (one of them may be its FD peer). Thus, the protocol cannot adapt to the FD communication directly.

Based on the CSMA/CA protocol [2], for the single-hop network model, the following studies have proposed some effective FD MAC protocols. The protocols proposed in [3,4] used the FD feature to increase their system throughputs. They extended the analytical model proposed in [2], and analyzed the collision probability of the FD node. Moreover, they fully considered the detection errors caused by the SI signal. In the HD networks, if more than one node sends frames, then the collision must occur. However, in the FD networks, when two nodes send frames simultaneously, the collision may not occur. For example, if they send the frames for each other, then both frames can be received correctly. Unfortunately, the collision probabilities solved in these protocols do not consider the actual communication mode. More importantly, they did not provide any mechanisms to coordinate the nodes to work in the FD modes. The authors in [5] extended the structures and semantics of the control frames Request-to-Send/Clear-to-Send (RTS/CTS). In addition to avoiding the transmission interferences, they can also initiate the FD communication between the nodes. However, at an idle time slot, the authors specified the transmission probability of each node and ignored that the new state transition caused by the FD communication may change its value. The protocol proposed in [6] has a similar problem.

In this paper, for this network model, we proposed an FD MAC protocol for the network layer to fully benefit from the FD mode. By using the FD features of the nodes and the cut-through mechanism, we reduced the channel access time wasted by the collisions and increased the FD opportunities in the network. The main contributions of the protocol are as follows:

First, we summarized the three scenarios where the frames are sent by the nodes in the network. We further discussed the protocol design requirements for each scenario.

Second, we provided the specific working process description of the protocol. After reading the information of the received frame’s header, the node can determine whether collisions exist and whether it can communicate with other nodes in the FD mode.

Third, we formulated the analytical model of the protocol. With reference to [2,7], we modeled the states (“active” and “passive” transmission, back-off) of each node and their transitions to a Markov chain. We solved the “active” transmission probability of the node and further solved the normalized throughput of the network.

Finally, we analyzed the throughput produced by our protocol in the numerical simulation. We also compared it with those of the CSMA/CA protocol used in the HD networks and an existing typical FD MAC protocol.

The rest of this paper is organized as follows. Section 2 builds up the system model. Section 3 describes the specific working process of the proposed FD MAC protocol and gives its analytical performance. Section 4 simulates and evaluates our protocol. Section 5 concludes the paper.

## 2. System Model

We assumed that there are *n* nodes in the network that all have FD capability and adopt the CSMA/CA mechanism to contend for the channel. Each node maintains *n*−1 frame sending queues for the other nodes and these queues are always non-empty. Each node can successfully receive signals from any of the other *n*−1 nodes, but it cannot simultaneously receive two or more signals from the others. This model is the single-hop network model, where no hidden terminal problems exist. For simplicity, we did not require the contention windows of the nodes to increase as the numbers of their transmission collisions increased. The contention windows of the nodes were all set to a constant value (denoted by *W*). If the node obtained a channel access opportunity, then it randomly selected one of the *n*−1 other nodes to send a frame. Here, the scheduling fairness [8] and routing protocol [9] were not considered. We assumed that no errors occurred in the frame transmission and each node could reduce the SI strength below the background noise. We did not discuss the interference cancellation in the physical layer [10]. The SI did not affect its own receiving or sensing. The frames sent by the nodes had the same size and were all composed of a header (abbreviated as “Hdr”) and a payload (abbreviated as “Pyl”). Their sizes and transmission times were denoted by *L_Hdr_* (*L_Pyl_*) and *T_Hdr_* (*T_Pyl_*), respectively. The semantic and size of each field of the frames were consistent with those used in the HD networks. When the node received a frame, we assumed that it could decode the frame’s header independently. This mechanism is called the cut-through [11], which is commonly used to fast-forward the frame (link layer) or packet (network layer).

## 3. Proposed FD MAC Protocol

According to the model above, in the HD networks, if more than one node sends signals, then collisions will occur. However, in the FD networks, two nodes are allowed to transmit signals simultaneously. Moreover, if two nodes in the network send the frames for each other, then the system throughput is twice that of the two nodes working in the HD mode. Hence, the proposed FD MAC protocol needs to increase this opportunity.

### 3.1. Three Scenarios for the Protocol

**Figure 1:**
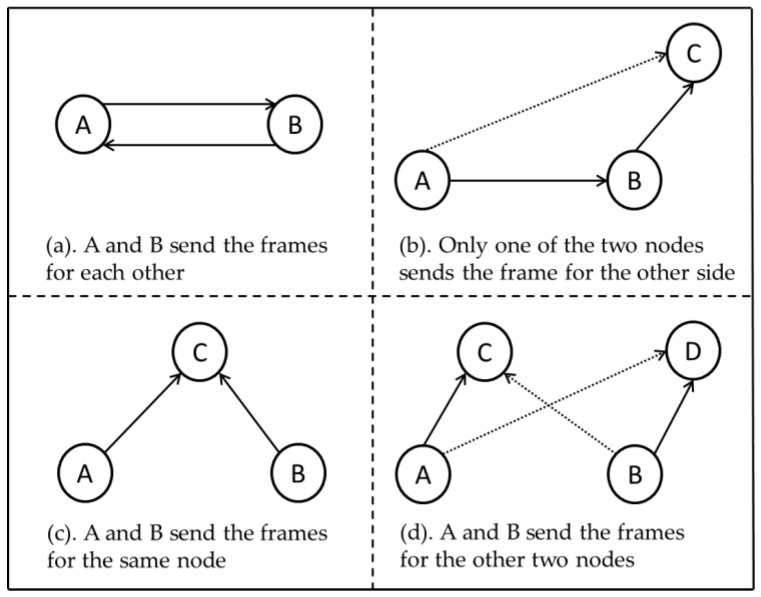
Four communication modes corresponding to the two nodes sending signals simultaneously.

The three scenarios where the frames are sent by the *n* nodes and their corresponding protocol design requirements are discussed as follows:

First, when only one sender sends the frame, the receiver can receive the frame in the HD mode correctly. No collisions will occur. However, to increase channel utilization, we should require the receiver to send a “reverse” frame to the sender at an appropriate time. Second, when three or more senders send their frames simultaneously, any node in the network will receive the transmitted signals from at least two other nodes. Regardless of how the protocol is designed, the collisions must occur. What is needed is to minimize the channel access time wasted by the collisions. Finally, when exactly two senders (A and B) send the frames simultaneously, they have the following four communication modes (Figure 1):

In Figure 1, the dashed line represents the interference signal, and the solid line represents the desired signal. As shown in Figure 1a, when the two nodes (A and B) send the frames for each other, the two frames can be received correctly. When only one of the two nodes sends the frame to the other side, as shown in Figure 1b, only one node (B) can receive the frame correctly. When the two nodes send the frames for the same node or for the other two nodes, as shown in Figure 1c,d, respectively, the two transmissions will interfere with each other and no receiver can receive the frame correctly. To this end, the protocol design can be divided into two cases. First, for the communication mode as shown in Figure 1a, we do not need to adjust the FD communication currently in progress. Second, note that the other communication modes either fail to exert the FD capabilities of the nodes (Figure 1b) or fail to produce effective throughput in the network (Figure 1c,d). Therefore, at an appropriate time, we should adjust them to the mode in which the two nodes send the frames for each other. For this problem, the MAC protocol proposed in [5] adds an “octal” field to the original RTS frame structure. The content of this field is a random number, which is used to compute a priority with the MAC address of the current node. When two nodes send the request frames simultaneously, the protocol can specify the FD communication according to this priority.

### 3.2. Protocol Description

**Figure 2:**
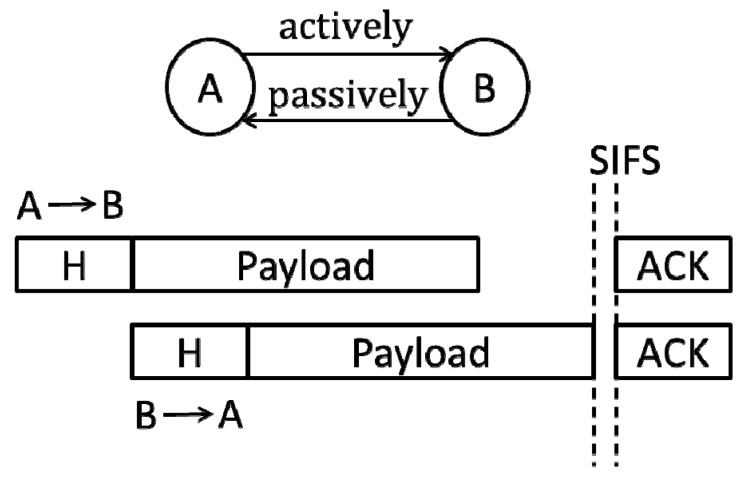
B obtains the MAC address of A from the received frame’s header and sends a frame to A (FD_1 mode).

In order to meet the protocol design requirements above, we describe the specific working process of the proposed FD MAC protocol in this section. We assumed that A obtains a channel access opportunity and selects B as the destination node to send a frame. During the transmission of the frame’s header, A always senses the channel to observe whether other transmitted signals exist. We discuss three possible conditions as follows:

First, A does not sense any signals. This condition means that no other nodes simultaneously send the frame’s header with A. For A, it only needs to continue sending the frame’s payload. For B, the protocol requires it to read the address of A in the received frame’s header and send a “reverse” frame to it. The transmission of the frame is “passive”. Many studies such as in [6,12] refer to it as the second transmission (ST). In contrast, when the back-off count of the node is reduced to zero, the “active” transmission of the frame is called the first transmission (FT). This communication mode (denoted by FD_1) is shown in Figure 2:

After B sends it over, it should move to the back-off states. Note that it can return to the original back-off count or reselect a back-off count. Currently, the MAC protocol standard for the FD communication does not clarify this problem. The FD MAC protocols proposed in recent years (e.g., [13,14]) basically consider the latter more reasonable. Thus, our protocol follows this mechanism.

Second, A senses the signals sent by the other nodes, but it cannot decode it. This condition means that at least two other nodes simultaneously send the frames’ headers with A. Note that these nodes also cannot decode any signals. The protocol requires all of them to terminate the transmissions of their frames’ payloads and move to the back-off states directly. In this case, no effective throughput exists in the network.

**Figure 3:**
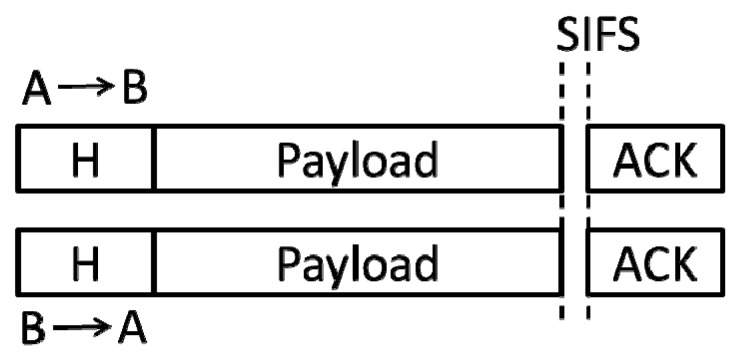
A and B send frames for each other simultaneously (FD_2 mode).

**Figure 4:**
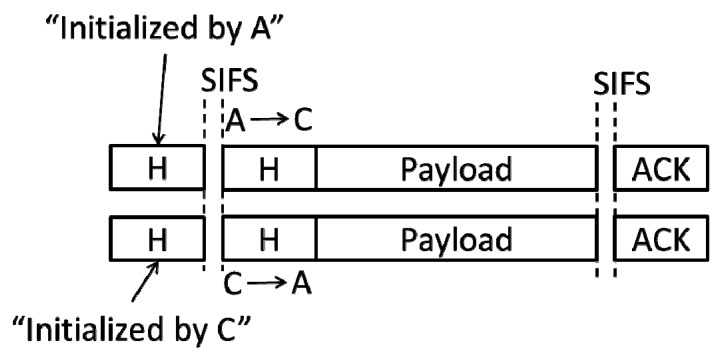
A and C re-conduct an FD communication.

**Figure 5:**
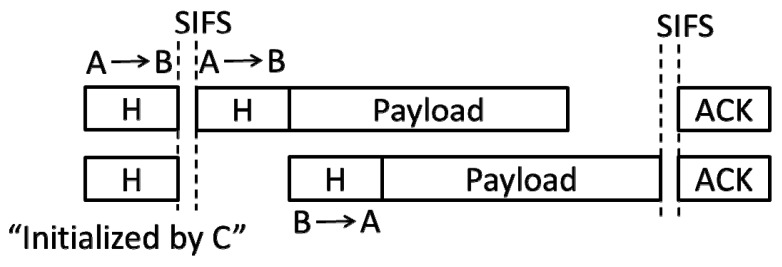
A sends the frame to B again and then B initiates the “passive” transmission (FD_3 mode).

Third, A senses the signals sent by other nodes, and it can decode it. This condition means that only one node sends the frame’s header with A simultaneously. If the frame exactly comes from B and its destination is A (the information obtained by B is consistent with this), then A and B only need to continue the transmissions of their frames’ payloads. This communication mode (denoted by FD_2) is shown in Figure 3:

Otherwise, A and this node (assuming it to be C) must communicate in one of the three modes as shown in Figure 1b–d. Here, the protocol can operate in two different methods. One method is that the protocol may require A and C to terminate the transmissions of the current frames and then send the new frames for each other [5]. That is, they re-conduct an FD transmission. This communication mode is shown in Figure 4:

The other method is that the protocol may require A and C to perform priority operations based on their addresses (or other metrics) (assuming the results of the two operations are consistent), to determine the node that can access the channel. If A has a higher access priority than C, then C moves to the back-off states directly and A sends the frame to B again. Subsequently, similar to the first case, B reads the MAC address from the frame’s header and then initiates the “passive” transmission. This communication mode is shown in Figure 5:

Clearly, this protocol operating method ensures that one of the two originally initiated transmissions can be implemented during the current contention period. Thus, we adopted this method. The communication mode is denoted by FD_3.

### 3.3. Modeling and Analyzing the Performance of the Proposed Protocol

According to the above protocol description, each node actually has a finite number of states. Moreover, each state is only related to the previous one. Hence, referring to the method in [2], we used a discrete Markov chain to model the states of each node and their transitions. After solving the steady states of the Markov chain, we further modeled the analytical performance of the protocol.

**Figure 6:**
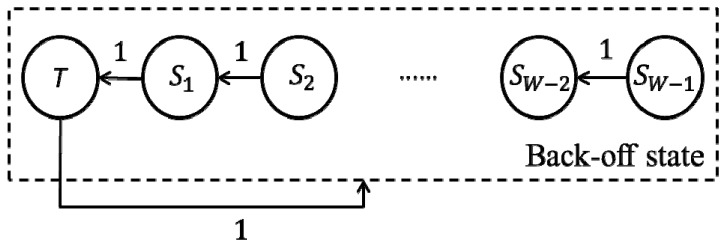
State transition diagram of the HD node in the network.

As a comparison, we first provide the state transition diagram of the HD node in the network [7], as follows:

In Figure 6, *S*_1_–*S_W_*_−1_ denote the back-off states, and *T* (also can be regarded as *S*_0_) denotes the transmission state. According to the CSMA mechanism, if the node senses that the channel is idle at the *t*−1 time slot, then it will reduce the back-off count at the *t* time slot. Otherwise, the back-off count remains unchanged. The actual time interval between the two consecutive back-off counts may be the length of a slot time (denoted by *σ*) or the length of the time required for a frame transmission. Let *t*→∞, [7] solves the steady probability of the node in the *T* state (we used *π_T_* to denote its probability, similarly hereinafter). That is, at any time slot, the transmission probability of the node is as follows:

(1) $$\pi_{T}=\frac{2}{W+1}.$$

In the HD networks, when two or more nodes in the *T* state are present at a time slot, the collisions will occur. *P_col(HD)_* denotes the collision probability. We also denote this using *T_col(HD)_*, the length of the time wasted by the collision. According to the value of *π_T_*, without using or using the control frames, [2] solves the system normalized throughputs in both cases, denoted by *Th_HD(basic)_* and *Th_HD(RTS)_*, respectively. In the physical sense, the throughput can be obtained from dividing the total number of the transmitted bits in a time interval (denoted by *L_tol_*; here “tol” is short for “total”) by the average length of the time interval (denoted by *L_ave_*; here “ave” is short for “average”).

**Figure 7:**
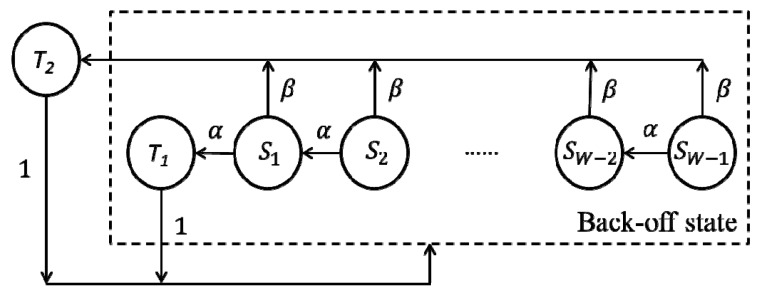
State transition diagram of the FD node in the network.

Then, we give the state transition diagram of the FD node in the network, as shown in Figure 7:

Note that each node has two transmission states (“active” and “passive”), which are denoted by *T*_1_ and *T*_2_, respectively. Here, the node can jump directly from the back-off states to the transmission state with probability *β*. Let *t*→∞, because *α* + *β* = 1, the steady probability of each state of the Markov chain in Figure 7 satisfies Equations (2) and (3).

(2) $$\pi_{S_{i}}=\alpha \pi_{S_{i+1}}+\frac{1}{W}\left(\pi_{T1}+\pi_{T2}\right),\begin{array}{cc} & i=0,\ldots ,W-2 \end{array},$$

where *π_S_*_0_ = *π_T_*_1_. For the last back-off state, we have:

(3) $$\pi_{S_{i}}=\frac{1}{W}\left(\pi_{T1}+\pi_{T2}\right),\begin{array}{cc} & i=W-1 \end{array}.$$

For *π_T_*_2_, we have:

(4) $$\pi_{T2}=\beta \sum_{i=1}^{W-1}\pi_{S_{i}}.$$

By Equations (2) and (3), the iterative expressions among each *π_Si_*(*i* = 1, …, *W* − 1) can be obtained as follows:

(5) $$\pi_{S_{i}}=\frac{1}{1+\alpha }\pi_{S_{i-1}},\begin{array}{cc} & i=W-1 \end{array},$$

(6) $$\pi_{S_{i}}=\left(1-\frac{\alpha^{W-i}}{\sum_{k=0}^{W-i}\alpha^{k}}\right)\pi_{S_{i-1}},\begin{array}{cc} & i=1,\ldots ,W-2 \end{array}.$$

According to the protocol description in Section 3.2, if only two nodes communicate in the FD_1 or FD_3 mode, then one of them can jump from the back-off states to the *T*_2_ state. In the FD_1 mode, the probability (denoted by *β*_1_) can be obtained as follows:

(7) $$\beta_{1}=\left(\begin{array}{c} n-1\\ 1 \end{array}\right)\pi_{T1}{\left(1-\pi_{T1}\right)}^{n-2}\frac{1}{n-1},$$

where the $\left(\begin{array}{c} n-1\\ 1 \end{array}\right)\pi_{T1}{\left(1-\pi_{T1}\right)}^{n-2}$ term represents the probability that only one other node (excluding the current node) in the network “actively” sends a frame, and the $\frac{1}{n-1}$ term represents the probability that the destination address of the frame is the current node. In the FD_3 mode, the solution of this probability (denoted by *β*_2_) needs to be discussed according to the communication mode of the two nodes. Basically, we can obtain the probability that they simultaneously send the frames, as follows:

(8) $$\beta_{2(basic)}=\left(\begin{array}{c} n-1\\ 2 \end{array}\right)\pi_{T1}^{2}{\left(1-\pi_{T1}\right)}^{n-3}.$$

First, in the mode as shown in Figure 1a, the two nodes initiate the transmission actively. We have *β*_2(1)_ = 0. Second, in the mode as shown in Figure 1b, we can obtain *β*_2(2)_ as follows:

(9) $$\beta_{2(2)}=\beta_{2(basic)}\cdot 2\left(\frac{n-2}{n-1}\frac{1}{n-1}\right)\cdot \frac{1}{2}\left(\frac{1}{n-2}\right),$$

where the $2\left(\frac{n-2}{n-1}\frac{1}{n-1}\right)$ term represents the probability that the two nodes communicate in this mode, and the $\frac{1}{2}\left(\frac{1}{n-2}\right)$ term represents the probability that one of the two nodes obtains the transmission opportunity and selects the current node as the destination. Third, in the mode as shown in Figure 1c, we can obtain *β*_2(3)_ as follows:

(10) $$\beta_{2(3)}=\beta_{2(basic)}\cdot \left(\frac{n-2}{n-1}\frac{1}{n-1}\right)\cdot \left(\frac{1}{n-2}\right),$$

where the $\left(\frac{n-2}{n-1}\frac{1}{n-1}\right)$ term represents the probability that the two nodes communicate in this mode, and the $\left(\frac{1}{n-2}\right)$ term represents the probability that the current node is selected as the destination by the two nodes. Four, in the mode as shown in Figure 1d, we can obtain *β*_2(4)_ as follows:

(11) $$\beta_{2(4)}=\beta_{2(basic)}\cdot \left(\frac{n-2}{n-1}\frac{n-3}{n-1}\right)\cdot \frac{1}{2}\left(\frac{1}{n-2}\right),$$

where the $\left(\frac{n-2}{n-1}\frac{n-3}{n-1}\right)$ term represents the probability that the two nodes communicate in this mode, and the meaning of the $\frac{1}{2}\left(\frac{1}{n-2}\right)$ term is consistent with the corresponding term in *β*_2(2)_. Furthermore, we can obtain *β*_2_ as follows:

(12) $$\beta_{2}=\sum_{i=1}^{4}\beta_{2(i)}.$$

Given that the node executes the “passive” transmission in the *T*_2_ state, according to the network model, it will not collide with others. When solving the collision probability of the network, we did not consider the probability that the node was in this state. Finally, we have the normalized condition for the probabilities of these states, as follows:

(13) $$\pi_{T1}+\sum_{i=1}^{W-1}\pi_{S_{i}}+\pi_{T2}=1.$$

According to Equations (5) and (6), the probability of each state can be converted to the expression of *π_T_*_1_. When the values of *n* and *W* are given, the value of *π_T_*_1_ can be solved by Algorithm 1. Here, we used *τ* to denote *π_T_*_1_.

 **Algorithm 1.** Solving the *π_T_*_1_ of each node **Require:**  The values of *n* and *W*; Initialize *i* = 0.0001; **while**
*τ* ≤ 1 **do**  *β* = *τ*(1 − *τ*)^*n*−2^; // here we use the expression of *β*_1_,        also we can use that of *β*_2_  *α* = 1 − *β*;  *g*(1) = 1 + *α*, *f*(1) = 1/*g*(1);  **for**
*i* = 2; I ≤ *W* − 1; *i*++ **do**    *g*(*i*) = *g*(*i* − 1) + *α^i^*;    *f*(*i*) = *α^i^*/*g*(*i*);  **end for**  *π_S_*_1_ = (1 − *f*(*W* − 1))*τ*;  **for**
*i* = 2; *I* ≤ *W* − 1; *i*++ **do**    *π_Si_* = (1 − *f*(*W* − *i*))*π*_*Si*−1_;  **end for**  *X* = (1 + *β*)sum{*π_Si_*}|_iϵ(1,*W*−1),*i*ϵZ_ + *τ*;  **if**
*X* − 1 < δ **then**    break;  **end if**  *τ* = *τ* + 0.0001; **end while**

Then, based on the value of *π_T_*_1_, we can solve the system normalized throughput (denoted by *Th_FD_*). At a time slot, 0, 1, 2, or more nodes may send frames. For different cases, the behaviors of the nodes and channel access times they occupy are different. To solve the average length of the time interval between the two consecutive back-off counts of the node, we need to analyze these cases respectively.

First, the probability that no node “actively” sends the frame (denoted by *P_idle_*) is as follows:

(14) $$P_{idle}={\left(1-\pi_{T1}\right)}^{n}.$$

The channel access time occupied by this case is exactly the length of a time slot (*σ*). Second, the probability that only one node “actively” sends the frame (denoted by *P_sgl_*; here “sgl” is short for “single”) is as follows:

(15) $$P_{sgl}=\left(\begin{array}{c} n\\ 1 \end{array}\right)\pi_{T1}{\left(1-\pi_{T1}\right)}^{n-1}.$$

In this case, the destination node will send a “reverse” frame after receiving the frame’s header. As shown in Figure 2, the corresponding channel access time is as follows (we ignored the propagation time of the signal, similarly hereinafter):

*T_sgl_* = DIFS + *T_Hdr_* + *T_Hdr_* + *T_Pyl_* + SIFS + *T_Ack_*. (16)

Third, the probability that two nodes “actively” send the frames simultaneously (denoted by *P_dbl_*; here “dbl” is short for “double”) is as follows:

(17) $$P_{dbl}=\left(\begin{array}{c} n\\ 2 \end{array}\right)\pi_{T1}^{2}{\left(1-\pi_{T1}\right)}^{n-2},$$

where the probability that the two are mutual destinations (denoted by *P_bi_*, here “bi” is short for “bidirectional”) is as follows:

(18) $$P_{bi}=P_{dbl}{\left(\frac{1}{n-1}\right)}^{2}.$$

As shown in Figure 3, the channel access time occupied by this case (denoted by *T_bi_*) is as follows:

*T_bi_* = DIFS + *T_Hdr_* + *T_Pyl_* + SIFS + *T_Ack_*. (19)

Moreover, the probability that the nodes communicate in the FD_3 mode is as follows:

*P_non-bi_* = *P_dbl_* − *P_bi_*. (20)

The channel access time occupied by this case (denoted by *T_non-bi_*) is as follows:

*T_non-bi_* = *T_sgl_* + SIFS + *T_Hdr_*. (21)

When more than two nodes “actively” send the frames simultaneously, the collisions will occur. The probability (denoted by *P_col(FD)_*) is as follows:

*P_col(FD)_* = 1 − *P_idle_* − *P_sgl_* − *P_dbl_*. (22)

Given that the collisions only waste the transmission time of a frame’s header, the channel access time occupied by this case (denoted by *T_col(FD)_*) is as follows:

*T_col_*_(*FD*)_ = DIFS + *T_Hdr_*. (23)

Furthermore, the average length of the time interval can be obtained as follows:

(24) $$T_{ave}=P_{idle}\sigma +P_{col(FD)}T_{col(FD)}+P_{sgl}T_{sgl}+P_{bi}T_{bi}+P_{non-bi}T_{non-bi}.$$

Moreover, the total number of the transmitted bits in a time interval can be obtained as follows:

(25) $$L_{tol}=2\times \left(P_{sgl}+P_{dbl}\right)\left(L_{Hdr}+L_{Pyl}\right).$$

Note that the length of the whole frame is considered in the solution of *L_tol_*. The subsequent numerical simulations for *Th_HD(Basic)_* and *Th_HD(RTS)_* are also based on this criterion.

Finally, the system normalized throughput can be obtained as follows:

(26) $${Th}_{FD}=\frac{L_{tol}}{T_{ave}}.$$

Note that we focused on the system throughput that could be achieved under the saturated flow. This performance metric can be directly converted to the transmission delay.

## 4. Numerical Simulation and Analysis

According to the discussion in Section 3.1, when each node in the network works in the FD and HD modes (the former adopts the proposed FD MAC protocol, and the latter adopts the traditional CSMA/CA protocol), we use different values of *n* and *W*.

**Figure 8:**
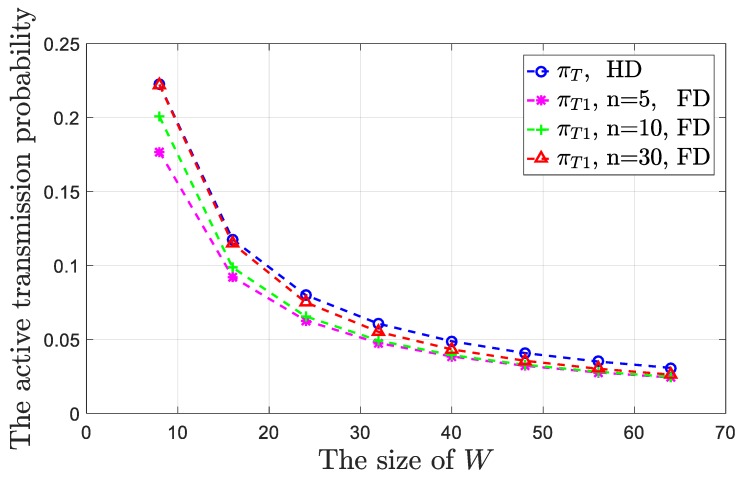
“Active” transmission probabilities for the proposed FD MAC protocol and the traditional CSMA/CA protocol versus the different values of *n* and *W*.

The values of *π_T_*_1_ and *π_T_* are shown in Figure 8.

As shown in Figure 8, in general, the largest difference between the values of *π_T_* and *π_T_*_1_ (in *n* = 5 and *W* = 64 case) was about 20%. As the size of *W* increased, both the values of *π_T_*_1_ and *π_T_* decreased because after a node sends the frame over, regardless of whether the transmission is successful, it will randomly select a back-off count in the contention window and enter the corresponding back-off state. According to the state transition diagrams as shown in Figure 6 and Figure 7, if the size of *W* increases, then the optional number of the back-off counts will increase and the node may select a larger back-off count. At this time, the “way” from the back-off states to the transmission state becomes longer. In other words, the probability that originally belongs to the transmission state is allocated to the back-off states. For the HD node, according to Equation (1), the value of *π_T_* is independent of *n*. However, for the FD node, when the size of *W* remains unchanged, the value of *π_T_*_1_ will increase as *n* increases. We used the *W* = 8 case for example. When *n* = 5, we have *π_T_*_1_ = 0.1768, but when *n* = 10, we have *π_T_*_1_ = 0.2005. This condition occurs because, according to the system model, the node which “actively” sends the frame randomly selects its destination among all the other nodes. Then, the two nodes communicate in FD_1 or FD_3 mode. At this time, a node will be present in the *T*_2_ state. According to Equations (7)–(11), if the value of *n* increases, then the probability (*β*_1_ and *β*_2_) that the node is selected as the destination will decrease. Furthermore, the node will be less likely in the *T*_2_ state (in the simulation, when *n* = 5, we have *π_T_*_2_ = 0.089, but when *n* = 10, we have *π_T_*_2_ = 0.0409) and more likely in the *T*_1_ state. Note that when *n* increases to a large value, the probability that the node is in the *T*_2_ state is very small. The value of *π_T_*_1_ approaches *π_T_*. In the simulation, when *n* = 30, the value of *β* (*β*_1_ + *β*_2_) equaled 6.17 × 10^−4^. Accordingly, the value of *π_T_*_2_ was only 4.8 × 10^−4^. When the size of *W* is large, although the value of *β* will decrease as *n* increases, given the probability that the node is in the back-off states increases and *π_T_*_2_ can be solved by *β* multiplying the sum of *π_Si_*(*i* = 1, …, *W* − 1), both the values of *π_T_*_2_ and the difference between *π_T_*_1_ and *π_T_* will increase.

When the communication modes as shown in Figure 1b–d appear in the network, if the protocol operates as shown in Figure 4, then neither of the two nodes initiates “passive” transmission, *β*_2_ does not exist, and *β* = *β*_1_. In fact, we lose the probability that the node jumps from the back-off states to the *T*_2_ state. The value of *π_T_*_2_ will decrease. Hence, the values of *π_T_*_1_ in such cases are slightly larger than those shown in Figure 8. In the simulation, when *n* = 5 and *W* = 8, here *π_T_*_1_ = 0.1841, but in the above case, *π_T_*_1_ = 0.1768. When we keep the value of *W* unchanged and only increase the value of *n*, the value of *π_T_*_1_ in such cases will be closer to *π_T_*. For simplicity, we did not provide a comparison of the values of *π_T_*_1_ in the two cases in detail.

The values of the parameters used in the simulation are shown in Table 1, which are consistent with those used in [2]. *L_RTS_*, *L_CTS_*, and *L_Ack_* denote the size of the RTS, CTS, and Ack frames, respectively. The first two are used in the traditional CSMA/CA protocol.

**Figure 9:**
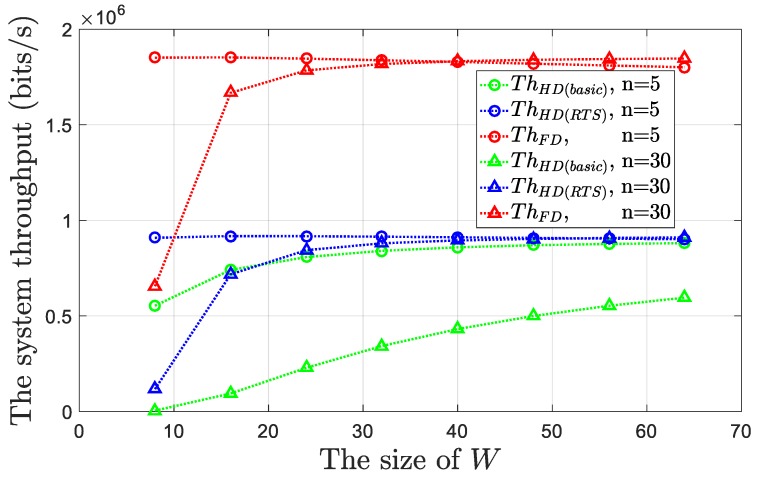
System throughputs for the proposed FD MAC protocol and the traditional CSMA/CA protocol versus the different values of *n* and *W*.

Subsequently, in Figure 9, we provided the numerical simulations for the performance of our proposed FD MAC protocol and compared it with that of the CSMA/CA protocol traditionally applied to the HD networks.

In general, regardless of the values of *n* and *W*, compared with the traditional CSMA/CA protocol, our protocol can significantly (at least twice) increase the system throughput. Basically, at a time slot, the “active” transmission probabilities of the nodes for the two protocols are similar. When only one node “actively” sends the frame, the latter produces twice as much throughput as the former. Furthermore, when two nodes “actively” send the frames, according to the traditional CSMA/CA protocol, a collision will occur. However, our protocol can coordinate the FD communication. For the proposed FD MAC protocol, we further discuss two details. First, as shown by the *Th_FD_* curve in Figure 9, when *n* = 5 and *W* > 40, the value of *Th_FD_* will decrease as *W* increases because when the size of *W* is large, the probability that each node is in the *T*_1_ state is low. If we have only a small value of *n*, then the network is more likely in the idle state. For example, when *n* = 5 and *W* = 64, the sum of *P_sgl_* and *P_dbl_* is only 0.1156, while *P_idle_* = 0.8843. Although the value of *T_idle_* is much smaller than the transmission time of the frames, according to Equation (26), the larger *P_idle_* and smaller *P_sgl_* and *P_dbl_* will also reduce the system throughput. Second, when the value of *n* is large and the size of *W* is small, the value of *Th_FD_* is low. This is because in these cases, contrary to the above, the network is more likely to be in the collision state. For example, when *n* = 30 and *W* = 8, we have *P_col(FD)_* = 0.9759, and the transmission collisions in the network waste considerable channel access time. Given that *P_col(FD)_* < *P_col(HD)_*, this problem is more prominent in the traditional CSMA/CA protocol. In this case, the value of *Th_HD(basic)_* is close to 0. Note that when the protocol uses the RTS/CTS mechanism, the system throughput will be significantly increased because although this mechanism cannot change the value of *P_col(HD)_*, it can reduce the channel access time wasted by the collisions from *T_Hdr_* + *T_Pyl_* to *T_RTS_*.

**Figure 10:**
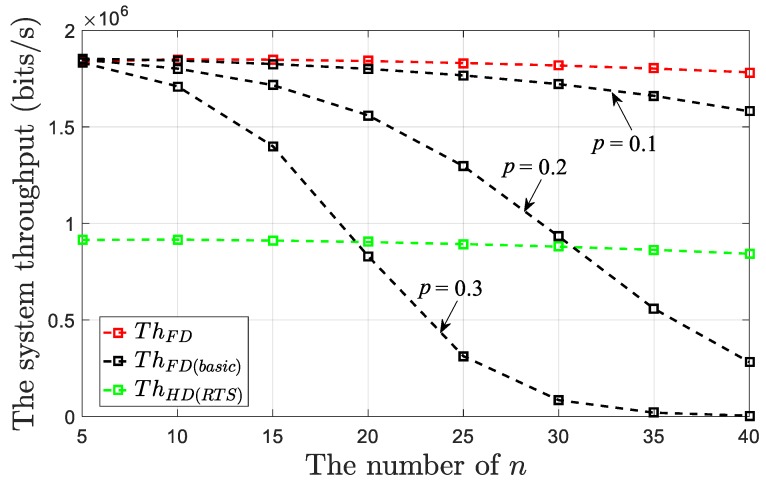
System throughputs for the proposed FD MAC protocol and the MAC protocol in [5] with fixed transmission probability versus the different value of *n* (*W* = 32).

Furthermore, in Figure 10, we kept the contention window of the nodes unchanged and compared the throughput produced through our protocol and that in [5] (denoted by *Th_FD_*_(*basic*)_) versus the different value of *n*. Here we assumed that *W* = 32. When *W* takes on other values, we have similar conclusions.

In [5], the contention mechanism of the nodes is *p*-persistent CSMA, where *p* is considered to be the “active” transmission probability at each time slot. Briefly speaking, when a node senses that the channel is idle, at the first time slot, it sends the frame with probability *p*. Even if it does not send the frame at this slot (with probability 1–*p*), the probability that it sends at the second time slot is still *p*, and so on. The nodes have no contention windows. Note that the value of *p* needs to be given in advance and it is difficult for each node to adjust this value adaptively. If the value of *p* is too small, then the channel cannot be fully utilized. If it is set at a too large value, then the number of collisions among the nodes will increase, resulting in reduced system throughput. As shown in Figure 10, when we set the value of *p* to 0.1, 0.2, and 0.3, respectively, ([5] sets this value to 0.2), *Th_FD_*_(*basic*)_ is reduced when compared with that of our protocol. Moreover, as *n* increased, the decrease of *Th_FD_*_(*basic*)_ will accelerate with the increase of the *p* value. When *p* is set to 0.2 or 0.3, as *n* increases to a certain extent (35 or 20 in Figure 10), *Th_FD_*_(*basic*)_ is even lower than *Th_HD_*_(*RTS*)_. We believe that the question of how to set an appropriate value of *p* is a key issue for this kind of work.

## 5. Conclusions

In single-hop wireless networks, using the FD features of the nodes can detect collisions while they are transmitting signals. The FD communication mode can improve the system throughput. In addition, the cut-through mechanism enables the nodes to decode the received frame’s header independently. Based on these three aspects, a MAC protocol was designed to reduce the collision occupancy time and increase the FD opportunities in the network. To ensure backward compatibility, the protocol requires the nodes to contend for the channel using the traditional CSMA/CA mechanisms. According to the working process, we modeled the states of each node and their transitions to a Markov chain. By solving the “active” transmission probability of the node, we obtained the analytical performance of the protocol. Simulation results showed that the system throughput produced by our protocol was at least twice that of the traditional CSMA/CA protocol used in the HD networks.

## Figures and Tables

**Table 1:** Value of each simulation parameter.

Parameter	Value
Frame payload	8184 bits
Frame header	272 bits
Channel rate	1 Mbps
ACK frame	112 bits
Slot time	50 us
RTS frame	160 bits
CTS frame	112 bits
SIFS	28 us
DIFS	128 us
